# Deep learning model accurately classifies metastatic tumors from primary tumors based on mutational signatures

**DOI:** 10.1038/s41598-023-35842-w

**Published:** 2023-05-30

**Authors:** Weisheng Zheng, Mengchen Pu, Xiaorong Li, Zhaolan Du, Sutong Jin, Xingshuai Li, Jielong Zhou, Yingsheng Zhang

**Affiliations:** 1 Beijing StoneWise Technology Co Ltd., Haidian District, Beijing, China; 2 Minzu University of China, Beijing, China; 3 Beijing University of Technology, Beijing, China; 4 Harbin Institute of Technology, Weihai, Shandong China

**Keywords:** Machine learning, Cancer genomics

## Abstract

Metastatic propagation is the leading cause of death for most cancers. Prediction and elucidation of metastatic process is crucial for the treatment of cancer. Even though somatic mutations have been linked to tumorigenesis and metastasis, it is less explored whether metastatic events can be identified through genomic mutational signatures, which are concise descriptions of the mutational processes. Here, we developed MetaWise, a Deep Neural Network (DNN) model, by applying mutational signatures as input features calculated from Whole-Exome Sequencing (WES) data of TCGA and other metastatic cohorts. This model can accurately classify metastatic tumors from primary tumors and outperform traditional machine learning (ML) models and a deep learning (DL) model, DiaDeL. Signatures of non-coding mutations also have a major impact on the model’s performance. SHapley Additive exPlanations (SHAP) and Local Surrogate (LIME) analyses identify several mutational signatures which are directly correlated to metastatic spread in cancers, including APOBEC-mutagenesis, UV-induced signatures, and DNA damage response deficiency signatures.

## Introduction

For most cancers, the metastatic spread is the main cause of cancer morbidity and mortality^1^. Studies have shown that nearly 90% of cancer deaths are the result of metastasis^2^. Once detaching from the primary site, metastatic cancer cells can travel through the circulatory system and establish themselves in new sites, potentially impacting multiple organs and systems^3,4^. The malignant cells can invade the local blood or lymphatic vessels to access to the systemic circulation. The lymphatic vessels transport immune cells throughout the body. Cancer cells can exploit these vessels to migrate to nearby lymph nodes and regional lymph nodes, evade the self-destruction meditated by immune cells, and eventually to distant organs and initiate tumorigenesis in those metastatic sites^5^. The distribution of metastatic sites for a given primary tumor is not arbitrary, but rather dictated by several factors such as the anatomical location, the origin of the cell, and molecular subtypes^6^. As a result, the identification of metastatic tumors and their origins is pivotal for choosing appropriate and effective therapeutic treatment in clinical practice.

Individuals accumulate somatic mutations over their lifetime as a result of various genetic and environmental factors^7,8^. These mutations can occur in any part of the genome and may have different effects on gene function and regulation. While the majority of these somatic mutations are neutral and accumulated in a passive manner, some genomic changes on the DNA sequences alter the regulation and function of genes, leading to the abnormal phenotypes of cells^7^. Accumulation of mutations in critical regulatory genes can lead to the development of diseases, such as cancers. Therefore, mutations serve as valuable features to decipher the developmental stages of tumorigenesis, including primary and metastatic tumors. By analyzing the pattern of mutations, researchers can gain insights into the genetic changes that occur during tumorigenesis.

Aberrant somatic mutations in DNA take many different forms, including single base substitutions (SBS), doublet base substitutions (DBS), small insertions and deletions (ID), and others. These genomic alterations are caused by a variety of mechanisms, including replication infidelity, spontaneous or enzymatic conversions and exposure to exogenous or endogenous mutagens^9^. Each of these mechanisms results in a distinctive pattern of mutations, known as mutational signatures^10^. Mathematical extraction of mutational signatures in large pan-cancer datasets revealed more than 100 different signatures, including SBS^11–13^, DBS^13,14^ and ID signatures^14^. Although the underlying mechanisms of many of these signatures are still unknown, some of them have been associated with established (e.g. smoking or UV radiation exposure) or novel (e.g. APOBEC mutagenesis) etiologies. Whole-Genome Sequencing (WGS) analysis of the metastatic solid tumors in pan-cancers has revealed certain specific patterns of mutational signatures in metastatic tumors^15^. Analysis of the matched primary and metastatic tumors further demonstrated the transformed profiles of mutational signatures from primary to metastatic tumors in colorectal^16^ and papillary thyroid cancers^17^. Additionally, based on SBS mutational signature, Sina Abdollahi, et al. were able to distinguish later-stage cancers from early-stage tumors using a feed-forward neural network^18^ based on SBS mutation signatures. This demonstrates the potential utility of utilizing the computational method as a diagnostic tool in cancer management, and the analysis of mutational signatures can provide valuable information about the progression of cancer.

**Figure 1:**
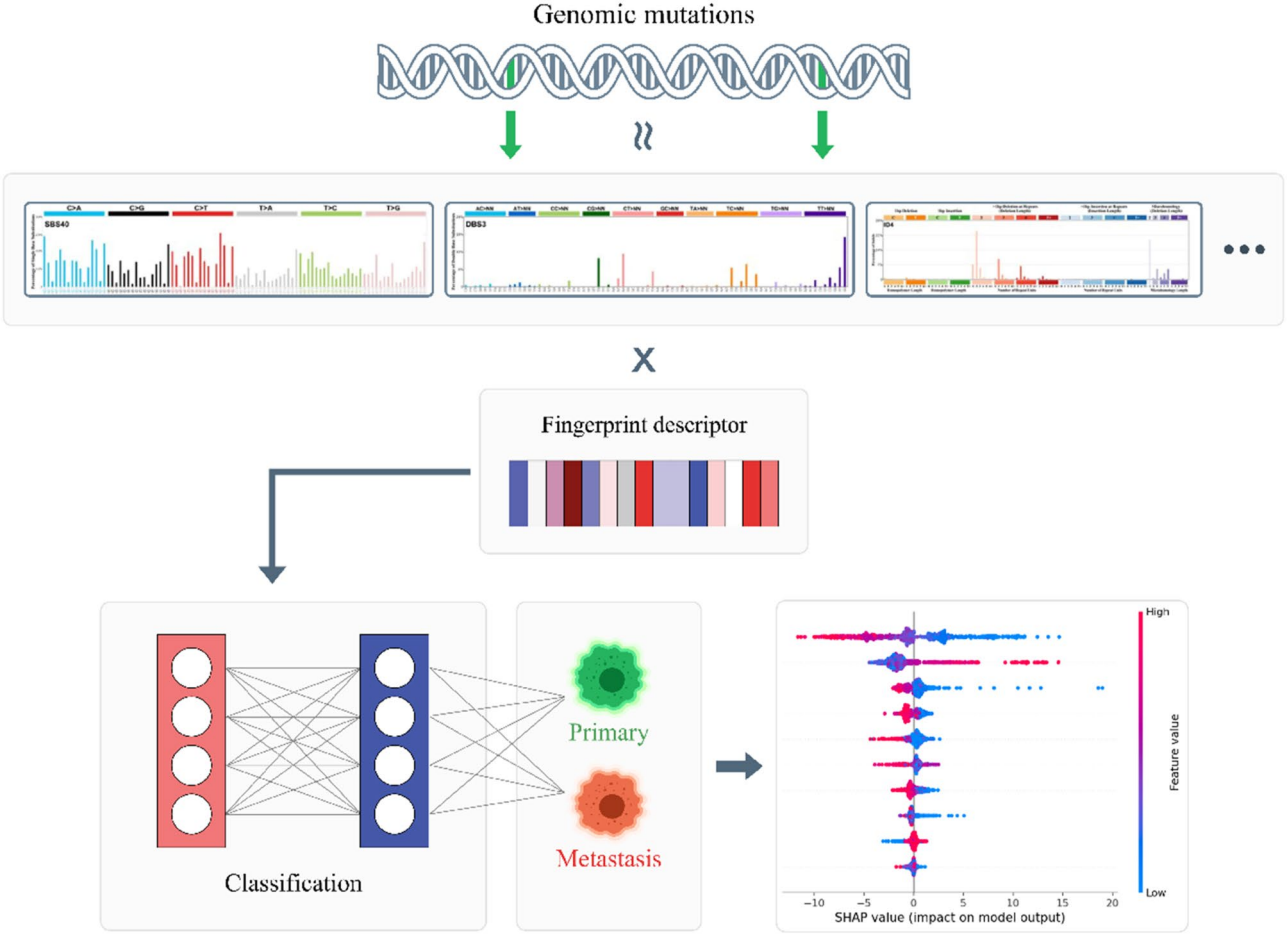
Illustraion of the workflow. Somatic mutations detected by WES or WGS data are characterized as SBS, DBS and ID mutational signatures and then the exposure of each signature in each sample is used as the input feature to the MetaWise model. MetaWise classify the sample as primary or metastatic tumors based on the mutational signatures fingerprint descriptor. Moreover, SHAP and LIME are applied to extract most informative signatures for the model.

In this study, we proposed a new approach, MetaWise, and suggested that SBS, in combination with DBS and ID mutational signatures in cancer genome, can serve as valuable markers in identifying primary and metastatic cancers. To support this hypothesis, we conducted mutational signature analysis of primary and metastatic tumors from TCGA pan-cancer studies^19^ and several other tumor cohorts^20–25^, which included more than 9000 primary and 1500 metastatic tumor samples. Our results indicate that incorporating SBS, DBS, and ID mutational signatures as features in the MetaWise model enhances its ability to accurately classify primary and metastatic tumors in the training, validation, and test sets (Fig. 1). Comparing to ML models and a DL model^18^, our model performs better in the identification of metastatic tumors from primary tumors with an averaged accuracy of 88.5% on the test sets. Validation of the model’s performance on different cancer types and external datasets demonstrates the generalizability of our model. Non-coding mutation signatures have a major impact on the models' performance. Furthermore, we extracted signatures that are the most informative in our model using SHAP^26^ and LIME^27^ methods. These signatures were found to play a significant role in the growth of cancerous metastatic cells, including those linked to APOBEC activity, DNA repair deficiency, and UV exposure. Our results demonstrate the potential utility of mutation signatures as markers to differentiate primary and metastatic cancers, which can aid in the development of better therapeutic strategies for cancer treatment.

## Results

### Mutational landscape of primary and metastatic tumors

**Figure 2:**
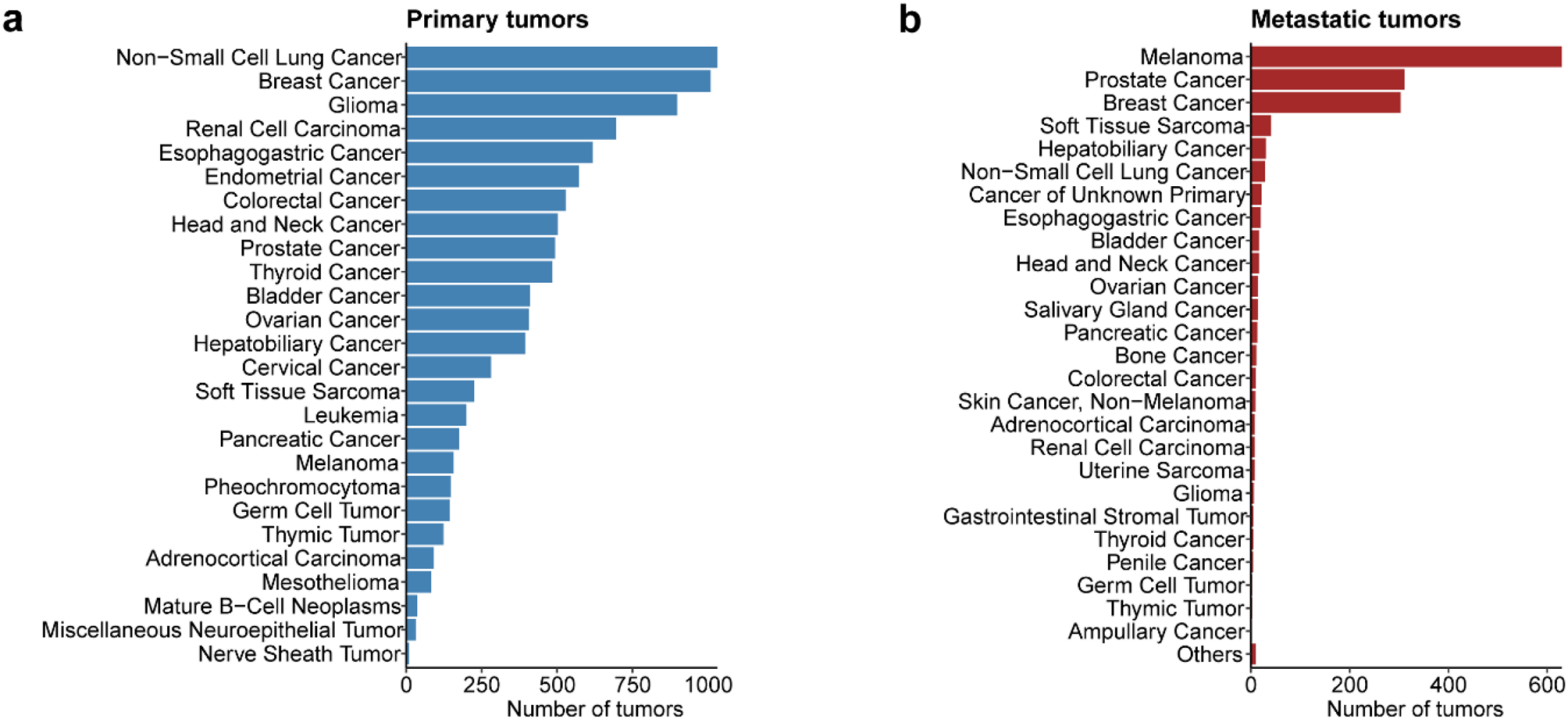
Cancer type distributions of primary and metastatic tumors. (**a**) The number of tumors in different cancer types of primary tumors from TCGA pan-cancer cohorts. (**b**) The number of metastatic tumors in different cancer types from TCGA and several other metastatic tumor cohorts.

The Cancer Genome Atlas (TCGA) is a landmark pan-cancer project containing somatic mutations, copy number variations, gene expression profiles, and other genomic characteristics among over 10,000 tumors from more than 30 different cancer types^19^. In order to study the mutational landscapes of primary and metastatic tumors, and to construct datasets for DL models, we retrieved the somatic mutations detected by WES from the TCGA project^19^ and several metastatic tumor cohorts, such as MET-500^23^, BRCA-igr^22^, SKCM-dfci^20^, and others^21,24,25^ from the cBioPortal database^28^. These datasets consist of more than 9700 primary tumors from more than 25 different cancer types and about 1500 metastatic specimens from more than 30 cancer types (Fig. 2a,b). The broad spectra of cancer types in these datasets allow us to train a more general and un-biased DL model for tumor stage classification.

**Figure 3:**
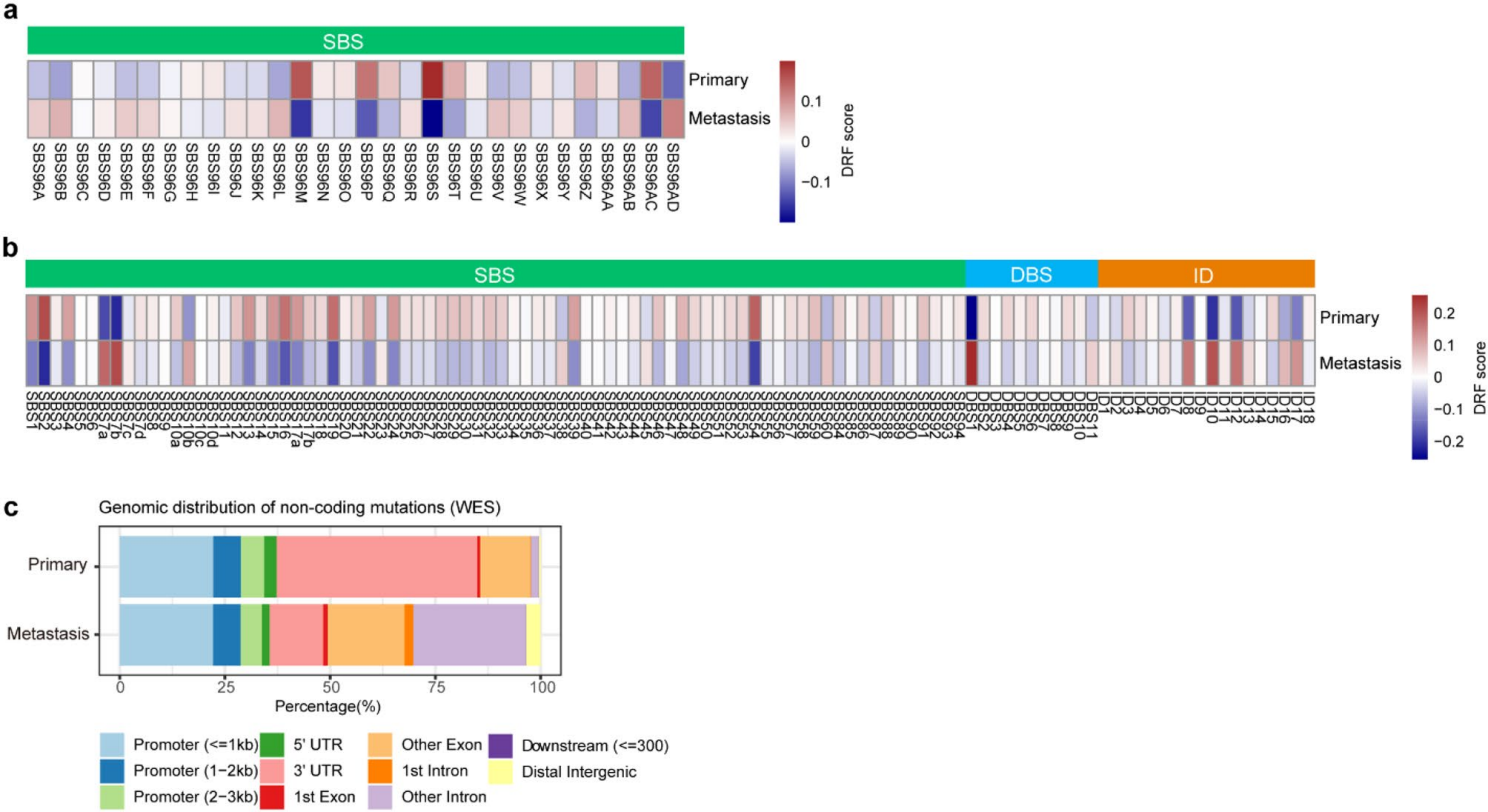
Mutation landscapes in primary and metastatic tumors. (**a**) The DRF results calculated from 30 de novo SBS signatures. (**b**) The DRF results calculated from 107 COSMIC mutational signatures. (**c**) The genomic distribution of non-coding mutations detected by WES data in primary and metastatic tumors.

To examine the mutational landscape, we conducted a mutational signature analysis of the somatic mutations of primary and metastatic tumors (Methods). Using the same procedures described in the previous study^18^, we extracted 30 de novo SBS mutational signatures from these data using SigProfiler^14,29^. The Catalog Of Somatic Mutations In Cancer (COSMIC) website has also curated 78 SBS, 11 DBS, and 18 ID mutational signatures, some of which associated with known etiologies (V3.3)^14^. We believe that inclusion of DBS and ID signatures to the datasets can provide extra information, and adopt COSMIC mutational signatures as references to calculate the exposure matrix to enhance the performance of the model. Applying the difference of relative frequency (DRF) analysis^18^ to the 30 de novo SBS mutational signatures and 107 COSMIC signatures profile, we revealed significant differences for some signatures between primary and metastatic tumors (Fig. 3a,b). The DRF analysis demonstrates that UV-induced signatures SBS7a and SBS7b as well as several ID signatures are enriched in metastatic tumors (Fig. 3b). To further examine the mutational signature profiles in different cancer types, we performed DRF analysis on three representing cancer types (melanoma, breast cancer, and prostate cancer). The analysis revealed that there were cancer type-specific enrichment patterns for mutational signatures, such as UV-related mutational signatures (SBS7a/7b, DBS1) being more enriched in metastatic melanomas and ID2 in metastatic prostate cancer. Additionally, some DBS and ID signature enrichments were consistent among different cancer types, for instance, ID10/ID11/ID12 being more enriched in the metastatic tumors in three cancer types. These results suggest that the difference of mutational signature distribution between primary and metastatic cancers could potentially serve as distinguishing features for cancer type classification (Supplementary Fig. S1).

### MetaWise model accurately classify metastatic tumors from primary tumors based on mutational signatures

The differential distribution of mutational signatures between primary and metastatic tumors lays the foundation that mutational signatures can be used as input characteristics in DNN models to classify metastatic tumors from primary tumors (Fig. 3a,b and Supplementary Fig. S1). To verify this, we constructed MetaWise, a ten-layer fully-connected DNN model that uses mutational signature profiles to classify metastatic and primary tumors using fivefold cross-validation for each dataset (Fig. 1). Due to the imbalanced population of primary and metastatic tumors, we randomly divided the primary tumors into 5 groups to correspond to the number of metastatic tumors. We selected this DNN architecture for our model due to the numerous advantages of DL models over traditional ML models, such as their capability to learn complex patterns, reduced need for feature engineering and improved accuracy, among others^30^. To examine whether our MetaWise model outperforms traditional ML models or the DiaDeL model in terms of predictive power in the classification of primary and metastatic tumors based on mutational signatures. We trained MetaWise, DiaDeL and four ML models (support vector machine (SVM), random forest (RF), XGBoost, and Logistic model) on the same dataset. The results demonstrate that MetaWise outperforms all ML models and DiaDeL with the highest F1 score, recall, AUC, and AUPRC (Table 1).

**Table 1:** Performance of ML models and DL models based on WES data with non-coding mutations.

Model	F1 score (%)	Recall (%)	AUC (%)	AUPRC (%)
SVM	45.5 ± 0.02	30 ± 0.01	64.2 ± 0.01	79 ± 0.03
RF	83.3 ± 0.02	77.9 ± 0.04	84.8 ± 0.01	89 ± 0.09
XGBoost	83.5 ± 0.05	80 ± 0.04	84.7 ± 0.06	88.4 ± 0.04
Logistic	81.1 ± 0.01	76.9 ± 0.05	82.6 ± 0.02	86.9 ± 0.02
DiaDeL	69.3 ± 2.27	64.5 ± 8.38	77.1 ± 0.27	84.4 ± 0.12
MetaWise	**86.1 ± 0.01**	**83.8 ± 0.03**	**86.8 ± 0.01**	**90 ± 0.01**

*SVM* support vector machine; *RF* random forest.

The highest value for each metric is shown in bold.

Our MetaWise model was trained on a pan-cancer dataset, which might allow us to study the generalizable metastatic patterns across different cancer types. Given the presence of imbalanced cancer types in the pan-cancer dataset, it is necessary to evaluate our model’s performance across different cancer types to test for potential bias towards certain cancer types. Firstly, we evaluated the performance of our pan-cancer MetaWise model (denoted as MetaWise-Pan) across various cancer types (Supplementary Table S1). MetaWise-Pan demonstrated prediction accuracies ranging from 73 to 100% across various cancer types, with the majority of cancers achieving an accuracy of 85% or higher. This indicates that the model possesses a certain level of pan-cancer prediction capability. It is interesting to note that the MetaWise-Pan model accurately predicted all primary and metastatic samples for head and neck cancer. For many cancer types, the metastatic tumors can be accurately predicted (with a high recall score). In cancer types with smaller sample sizes, metrics such as F1 score, AUC and AUPRC were more prone to fluctuate due to the influence of sample size (Supplementary Table S1). Overall, the measurements were comparable across different cancer types, revealing the model’s generalization potential.

In different cancer types, particularly those with fewer samples, some primary samples were misclassified as metastatic samples by MetaWise-Pan (with higher MetaWise-Pan prediction score). Since all primary samples in the testing dataset are from TCGA and include detailed clinical information, we compared the clinical outcomes between the primary samples with higher MetaWise-Pan prediction scores (HMPS, misclassified as metastatic tumors) and those with lower model prediction scores (LMPS, accurately classified as primary tumors). As depicted in Supplementary Fig. S2, patients in the HMPS group exhibited significantly lower disease-specific survival compared to those in the LMPS group (p = 0.045, log-rank test). This finding suggests that primary tumors with a high MetaWise-Pan prediction score are more prone to progression, indicating a poorer clinical outcome for the patients. We hypothesize that the MetaWise-Pan model prediction scores in primary tumors might be associated with clinical outcomes of the corresponding patients.

To test the feasibility of using MetaWise as a classifier for a specific cancer type, we further trained, validated, and tested it in a cancer type-specific manner for three representative cancer types: melanoma, breast and prostate cancers. The mutational signatures detected in each cancer type were also filtered by three thresholds (2.5%, 5% or 10%) to study the influence of signature detection procedure and remove potential noise. The results showed that the performance of the MetaWise-Mel model improved with increasing filtering thresholds, with the highest performance achieved at a 10% cutoff, comparable to the model without threshold-based filtering. For breast cancer and prostate cancer, the model’s performance is better at a 5% cutoff compared to 2.5% and 10% cutoff, and the performance at the 5% threshold is also similar to the model without threshold-based filtering. This suggests that setting a 5% cutoff may effectively balance the signal-to-noise ratio in the mutational signature detections for breast cancer and prostate cancer (Supplementary Table S2). We compared the performance of three specific cancer models and the pan-cancer model on the corresponding cancer-specific data. The performance of three specific cancer models was better on the cancer specific data, particularly for breast cancer and prostate cancer. These findings demonstrate that our proposed architecture could leverage the advantages of datasets that are tailored to specific cancer types, instead of using imbalanced data cross various cancer types. The performance on melanoma model dropped a little compared to the pan-cancer model, while the overall performance of the MetaWise model constructed on a specific cancer type with hundreds of samples was lower than that of the model constructed on the pan-cancer datasets. These results may imply that the pan-cancer model could achieve better performance with a properly balanced data across cancer types and a larger data size.

The use of independent datasets can help determine the generalizability of the approach. We acquired two additional WES cohorts (prostate cancer: pcbm_swiss^31^ and skin melanoma: skcm_yale^32^) from publicly available datasets to test the generalizability of our model. As shown in results (Supplementary Table S3), the model’s performance is comparable to that of the cancer type-specific model, with an accuracy of approximately 74% for the cancer type-specific model and 70–75% for the pan-cancer model. These results indicate that our model is generalizable and applicable to diverse datasets.

In addition, assessing the performance of the MetaWise model in patients with paired primary and metastatic tumors is critical and informative. To this end, we obtained WES data from primary and metastatic tumors in 9 treatment-naïve breast cancer patients^33^. Our results indicate that the MetaWise model can accurately classify paired primary and metastatic tumors in 6 out of 9 patients. For the remaining 3 patients, the primary and metastatic tumor samples could not be accurately classified. We observed that 2 of these 3 cases (Case5 and Case6) had a low mutation count (< 50 mutations per sample) in their WES data, which may have contributed to an increase in the sum-squared error (SSE) in the assignment of mutational signatures (Supplementary Table S4).

### Non-coding mutations impact model performance

Recently, non-coding mutations have received significant attention for their role in tumorigenesis^34,35^. Wei Jiao et. al have developed a DNN model that can accurately classify different types of cancer based on somatic passenger mutations, most of which are non-coding mutations^36^. It is intriguing to determine if these non-coding mutations have any impact on the mutational signature profile and the accuracy of primary and metastatic tumor classification. The WES datasets contain some non-coding mutations, such as those found in the untranslated regions (UTRs), introns, and other regions (Fig. 3c). To access the influence of these mutations, we excluded the non-coding mutations from the datasets, and conducted mutational signature analysis as described above. We also evaluated MetaWise based on the mutational signature profiles with and without non-coding mutations. The results reveal that MetaWise has improved performance when using the datasets that include non-coding mutations, regardless of whether 30 de novo SBS signatures or 107 COSMIC signatures were used (Table 2). To further investigate whether the influence of non-coding mutations is biologically relevant or just because of the additional number of mutations, we conducted an experiment based on two datasets: one the complete removal of non-coding mutations, and the other randomly removal of the mutations equal to the number of non-coding mutations in each sample (Supplementary Table S5). We found that MetaWise performed worse on datasets without non-coding mutations comparing to the randomly removed sets, thus highlights the significance of non-coding mutations in mutational signatures and the accuracy of the DNN model.

**Table 2:** The performance comparison between MetaWise and DiaDeL on datasets with and without non-coding mutations.

Dataset	# of features	Model	Val acc (%)	Test acc (%)	F1 score (%)	Recall (%)	AUC (%)	AUPRC (%)
WES.wt.nc-107	107	MetaWise	**89.29**	**88.5 ± 0.03**	**86.1 ± 0.01**	**83.8 ± 0.03**	**86.8 ± 0.01**	**90 ± 0.01**
WES.wt.nc-107	107	DiaDeL	78.5	77 ± 0.27	69.3 ± 2.27	64.5 ± 8.38	77.1 ± 0.27	84.4 ± 0.12
WES.wt.nc-30	30	MetaWise	76.3	73.4 ± 0.09	75.1 ± 0.06	77 ± 0.09	75.5 ± 0.06	80.7 ± 0.03
WES.wt.nc-30	30	DiaDeL	73.60	72.1 ± 0.02	60.4 ± 1.92	54.5 ± 8.04	72.8 ± 0.15	81.1 ± 0.1
WES.wo.nc-107	107	MetaWise	85.06	82.2 ± 0.18	80 ± 0.17	78 ± 0.05	81.1 ± 0.03	85.3 ± 0.03
WES.wo.nc-107	107	DiaDeL	72.3	74.2 ± 0.18	60.1 ± 2.5	51.6 ± 6.8	73.8 ± 0.22	81.5 ± 0.1
WES.wo.nc-30	30	MetaWise	70.78	73.1 ± 0.15	67.8 ± 0.03	63.5 ± 0.1	70.8 ± 0.03	77 ± 0.03
WES.wo.nc-30	30	DiaDeL	70.3	68.3 ± 0.33	49.7 ± 2.86	38.6 ± 5.75	69 ± 0.28	79.9 ± 0.1

“WES.wt.nc” represents for “WES data with non-coding mutations” and “WES.wo.nc” represents for “WES data without non-coding mutations”, respectively. 107 and 30 represent the 107 COSMIC mutational signatures and 30 De novo signatures used as the feature sets in DL models. Highest value of each metric is shown in bold.

To evaluate our model against the state-of-the-art model, we built the DiaDeL model based on the same datasets above. Both DiaDeL and MetaWise yield significantly better results when the mutational signatures include non-coding mutations, as compared to those without non-coding mutations (Table 2). Furthermore, MetaWise outperforms DiaDeL in the same datasets with the same features. MetaWise based on the 107 COSMIC signatures with non-coding mutations has the best performance (Table 2 and Supplementary Table S5, with an accuracy of 88.5%, a F1 score of 86.1%, a recall of 83.8%, an AUC of 86.8% and an AUPRC of 90%), showing the strength of this DNN model when incorporating both coding and non-coding region SBS, DBS, and ID mutational signatures.

WGS technique detects genomic alterations on a whole genome scale, thus most of the alterations in the non-coding regions can be revealed. To further investigate the impact of non-coding mutation signature on model performance, we studied the somatic mutations detected by WGS from the Pan-Cancer Analysis of Whole-Genome (PCAWG) project^37^ and a metastatic tumor cohort called POG570^38^, including approximately 1138 primary and 266 metastatic tumors in breast cancers, ovarian cancers, prostate cancers, and so on. We randomly selected equal number of primary and metastatic tumors from the same cancer types and constructed the WGS dataset to test our model. The results show that MetaWise model exhibited superior performance on “WGS non-coding” data, compared to “WGS coding” data, demonstrating that the non-coding data in the WGS significantly affected the accuracy of the model (Table 3). It is also noteworthy that MetaWise also performed better on “WGS non-coding” data than on “WGS all data” (containing both coding and non-coding mutations). This observation might indicate a potential negative impact of coding mutations on the model’s predictive power. Indeed, in Wei Jiao’s DNN model^36^, the incorporation of driver mutations (most of which are coding mutations) resulted in a reduction in model performance. To further demonstrate the significance of non-coding mutations, we conducted a similar experiment as WES data. We randomly selected mutations equal to the number of non-coding mutations in each sample, and evaluated the performance of MetaWise on those datasets, the results reveal that the model based on WGS non-coding datasets performed better than that on WGS random mutation datasets, emphasizing the specific improvement of non-coding mutations to the model’s performance (Table 3).

**Table 3:** Performance of MetaWise on WGS datasets.

Dataset	Test acc (%)	F1 score (%)	Recall (%)	AUC (%)	AUPRC (%)
WGS_coding_107	68.2 (± 0.93)	60.2 (± 1.36)	63.1 (± 1.74)	67.2 (± 1.03)	67.5 (± 0.85)
WGS_coding_89	72.7 (± 0.03)	66.2 (± 0.09)	70.2 (± 0.27)	72.3 (± 0.05)	72.2 (± 0.05)
WGS_non-coding_107	80.9 (± 0.11)	77.5 (± 0.12)	**85.7 (± 0.11)**	81.8 (± 0.09)	81 (± 0.08)
WGS_non-coding_89	**86.4 (± 0.04)**	**85.3 (± 0.03)**	83.7 (± 0.25)	**86.2 (± 0.04)**	**89.5 (0.05)**
WGS_random_nc_107	79.1 (± 0.24)	69.5 (± 0.7)	64.3 (± 1.87)	76.3 (± 0.39)	78.1 (± 0.3)
WGS_random_nc_89	81.4 (± 0.35)	80.1 (± 0.42)	79.8 (± 0.77)	81.3 (± 0.35)	85.2 (± 0.21)
WGS_all_107	80 (± 0.12)	75.3 (± 0.07)	79.8 (± 0.95)	80 (± 0.05)	80.4 (± 0.1)
WGS_all_89	82.7 (± 0.24)	82.1 (± 0.27)	83.7 (± 0.4)	82.8 (± 0.24)	86.1 (± 0.15)

The highest value for each metric is shown in bold.

107 and 89 represent the 107 COSMIC mutational signatures and 89 COSMIC mutational signatures without sequencing artefacts signatrues.

### Feature selection to interpret the MetaWise model

**Figure 4:**
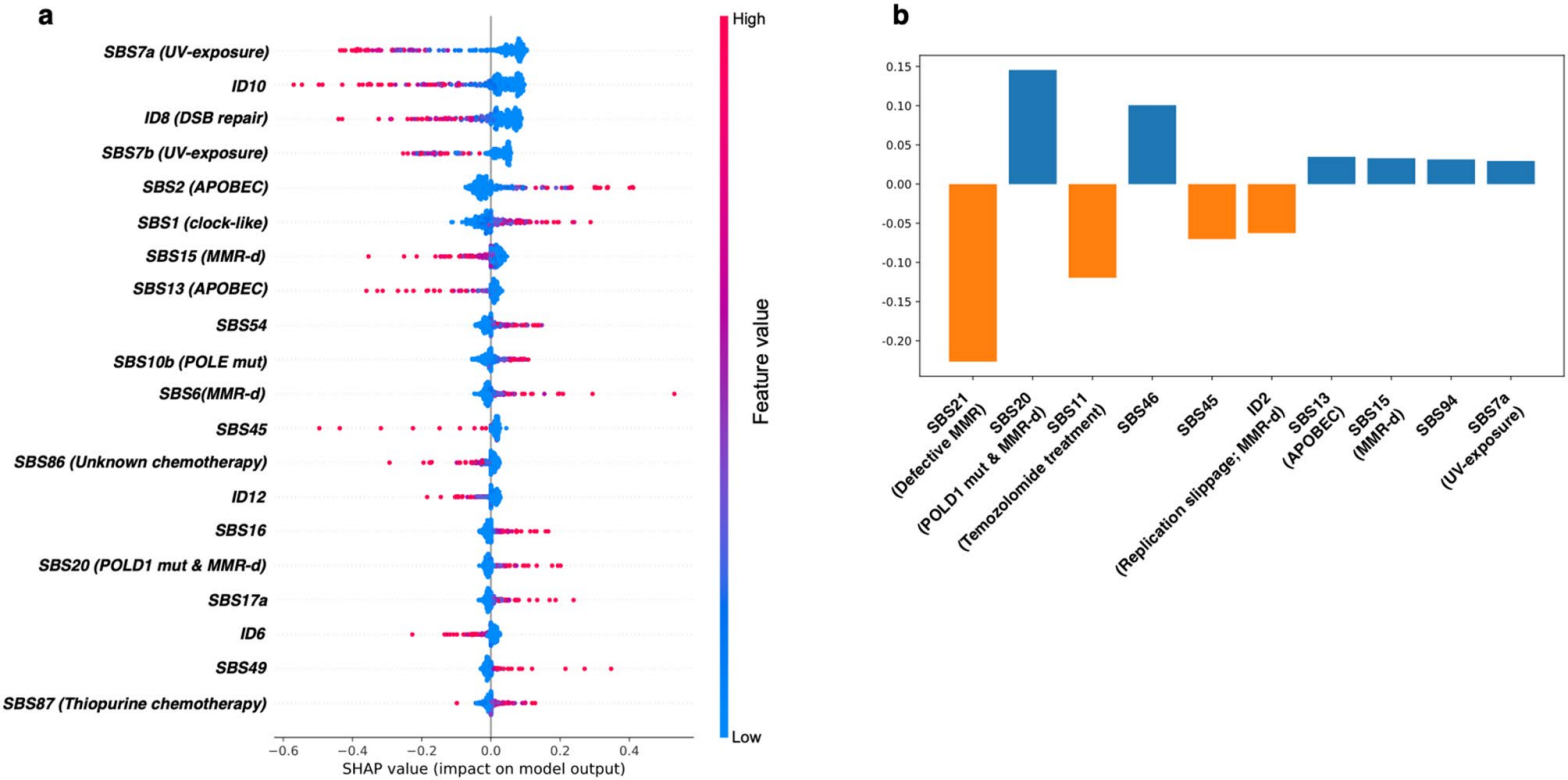
Interpreting the MetaWise model with the framework of SHAP and LIME. (**a**) Most informative mutational signatures selected by SHAP. The ranking of the signatures is based on the average absolute SHAP value, representing the most important feature for the model. Each dot represents a sample, and the color ranging from blue to red denote the SHAP values from low to high. A more dispersed sample distribution implies a greater impact of the signature, with the x-axis representing the positive or negative influence of the sample’s SHAP value. Samples that are both red and have larger positive or negative SHAP values represent a greater positive or negative impact. (**b**) Most informative mutational signatures selected by LIME. Signatures with positive values impact positively on the model’s prediction, while signatures with negative values impact negatively on the model’s prediction.

DRF analysis of mutational signature distribution revealed the significant differences of some mutational signatures between primary and metastatic tumors (Fig. 3a,b). These differences are likely to give the distinguishing power to the MetaWise model. To decipher which mutational signatures contribute to the performance of the model, we applied two methods, SHAP and LIME, to explain the prediction power of models. The SHAP analysis performs hyper-parameter tuning and feature selection simultaneously in a single pipeline^26^, while LIME analysis performs feature selection via training local surrogate models^27^. Although they share the goal of providing interpretable explanations, they have different underlying principles and algorithms, which can result in differences in their outputs. SHAP is based on the idea of Shapley values from cooperative game theory and aims to explain the contribution of each feature to the prediction by considering all possible combinations of feature values. The SHAP values are unique and represent a fair allocation of the prediction among the features. On the other hand, LIME generates local explanations by fitting a simple, interpretable model to the prediction of the original model in the vicinity of a specific instance, which provide an inherently local faithful approximation of the original model's behavior. We employed both methods in an attempt to produce a more comprehensive analysis outcome from various perspectives, and we believe that the overlap between the results of the two methods and the greater proportion of the specific results can offer more valuable insights. The results of SHAP show that DNA repair deficiency signatures (SBS15/SBS6/SBS20, etc.), APOBEC associated signature (SBS2/SBS13), UV-light induced signatures (SBS7a/SBS7b), and signatures associated with chemotherapy (SBS86/SBS87) are the most informative mutational signatures for our MetaWise model (Fig. 4a). LIME analysis showed that DNA repair associated signatures (SBS21/SBS20/SBS15, etc.), APOBEC associated signature SBS13, UV-associated signature SBS7a, and chemotherapy signature SBS11 among others are the most informative signatures for the classification of primary and metastatic tumors (Fig. 4b). Although, there are some differences between SHAP and LIME, they both captured signatures associated with similar etiologies. For example, APOBEC signatures, DNA repair deficiency signatures, UV-exposure signatures, and chemotherapy signatures were both revealed by SHAP and LIME. It’s been reported that APOBEC mutagenesis is associated with tumor evolution and heterogeneity in metastatic thoracic tumors^39^. Additionally, UV-radiation induced inflammation promotes the metastasis in melanoma^40^. Furthermore, DNA damage response deficiencies, such as defective of homologous recombination DNA damage repair and defective of DNA mismatch repair, have been reported to be enhanced in the brain metastasis of colorectal cancer^16^. Taken together, these results demonstrate these mutational signatures have significant contribution to the metastatic events in cancers.

To investigate the role of mutational signatures in various cancer types, we conducted SHAP and LIME analyses on our three cancer-specific models. Our findings reveal that certain signatures, such as the mismatch repair deficiency signatures (SBS6/SBS15/SBS20), APOBEC-associated signatures (SBS2/SBS13), and chemotherapy signatures (SBS87/SBS32) are shared across different cancer types. Moreover, we identified cancer-specific signatures that reflect distinct mutational processes involved in the development of each cancer type, for instance, UV-exposure associated signature DBS1 is significant in melanoma, SBS12/SBS16/ID16 are in breast cancer, while Aflatoxin exposure signature SBS24 and indirect effects of AID signature (SBS85) are highly informative in prostate cancer (see Supplementary Fig. S3).

To further investigate the influence of mutational signatures at different mutational signatures detection thresholds, we conducted SHAP and LIME analyses on the three cancer-type-specific models trained with mutational signatures detected with various filtering thresholds. The results showed that across different cancer-type-specific models, there is consistency in the top influencing mutational signatures at various thresholds (Supplementary Fig. S4-S6). For instance, in melanoma, a significant number of UV signatures (SBS7a/SBS7b/SBS38/DBS1) consistently rank top at different thresholds. In breast cancer, APOBEC signatures (SBS2/SBS13), clock-like signature SBS1 and DNA repair-associated signatures consistently appear among the top influencing signatures. In prostate cancer, deficiency in DNA mismatch repair signatures, chemotherapy-related signatures (SBS87) and the Aflatoxin signature (SBS24) consistently demonstrate substantial contributions, suggesting the need for further investigation into the potential influence of the Aflatoxin in prostate cancer. In breast cancer, the impact of sequencing artefacts signatures (SBS50/SBS58) can be effectively mitigated under all three threshold conditions. In melanoma and prostate cancer, although the influence of sequencing artefacts signatures persists under different thresholds, their impact on the model’s decision decreases as the threshold increases. This observation suggests that such filtering steps may help to alleviate the noise effects in the mutational signature extraction procedure.

## Discussion

To develop DNN models that can distinguish between metastatic and primary tumors, our study demonstrated that combining SBS with DBS and ID signatures as input features significantly improved the model performance. In comparison to SBS signatures, DBS and ID signatures are less well-studied due to their low frequency in cancer genome and limitations in characterization methods. However, with the advancement of high-throughput sequencing in cancer genomes, numerous DBS and ID signatures have been identified^13,14^. The genomic distribution and sequence compositions of DBS and small IDs are non-random^11,12^ and associated with known mutational processes^13,14^. Therefore, DBS and ID signatures can provide insightful biological information to the models. Recently, two studies by Ruben et al.^41^ and Christopher D Steele, et al.^42^ have introduced two types of characterization of chromosomal instability (CIN) signatures through pan-cancer studies. The CIN has also been linked to metastasis through the cGAS-STING cytosolic DNA-sensing pathway^43^. In future work, it could be beneficial to include CIN signatures in models predicting metastatic tumors.

Our results showed that including non-coding mutations in the WES data's mutation profiles enhances the characterization of mutational signatures and improves model performance. Most of the non-coding mutations detected by WES data are located in the key regulatory regions, such as promoters and UTRs. Interestingly, there is a higher proportion of mutations occurred in the 3’ UTR regions in primary tumors than metastatic tumors (Fig. 3c). Those regulatory elements, such as miRNA, regulate gene function by targeting the 3’ UTR regions of the mRNA transcripts^44^. The somatic mutations in these regions might affect the post-transcriptional regulation of key regulatory genes, leading to tumorigenesis and metastatic spread of cancers^45,46^. Although we were unable to test WGS data on larger scale due to the data confidentiality limitations, the performance of the model still showed significant improvement when a small amount of WGS data was used, highlighting the importance of non-coding mutation profiles and the need for access to larger cohorts of WGS data.

The DRF analysis revealed significant differences in the distribution of mutational signatures between primary and metastatic tumors, such as UV-induced signatures and others. Through the SHAP and LIME analysis on the MetaWise model, more informative combinations of mutational signature for distinguishing metastatic from primary tumors were extracted and aligned well with the results of the DRF analysis. APOBEC-induced signatures, UV-induce signatures, and DNA damage response deficiency signatures are the most crucial mutational processes in tumor metastasis^39,40^, which deserve further investigation in the transition process from primary to metastatic tumor. From the SHAP and LIME analyses of Pan-cancer model or cancer-type-specific models, multiple signatures possible be sequencing artefacts are detected frequently. To address this issue, we calculated the percentage of samples with these artifactual signatures in cohort-wise (Supplementary Fig. S7). We did find some specific enrichment patterns of artifactual signatures in specific cohorts. For instance, SBS45/SBS49 were specifically enriched in TCGA-SKCM cohort (containing both primary and metastatic samples), and the SHAP/LIME analyses of MetaWise-MEL selected those two signatures as top influential signatures. This might lead to the decrease classification power of MetaWise-MEL model and indicating the necessary of removing those signatures as input features for the models.

## Methods

### Data selection

The somatic mutations from the WES dataset of TCGA and other metastatic cohorts were obtained from the cBioPortal database (https://www.cbioportal.org/)^28^ using the cBioPortalData packages within the R environment^47^. The WES data were re-constructed into two datasets, one with all somatic variants in both coding and non-coding regions (referred to as “WES with non-coding”), and another one with only those variants detected in the coding regions (referred to as “WES without non-coding”). These two datasets were applied to investigate the influence of non-coding variants on model performance.

The somatic mutations from the WGS data in PCAWG37 and POG570 cohort^38^ were obtained from https://dcc.icgc.org/pcawg and https://www.bcgsc.ca/downloads/POG570/, respectively. The WGS data consists of more than 1138 primary tumors and about 266 metastatic tumors in breast, pancreatic, ovarian, prostate, and other cancers. To eliminate the impact of cancer types on the model performance, we randomly extracted approximate primary data while keeping the cancer type distribution of the primary data consistent with that of metastasis (referred to as “WGS_all”). Moreover, to understand the impact of mutations in the coding and non-coding regions on the model, we also generated separate datasets for coding region-only mutations, non-coding region-only mutations, and randomly selected mutations equal to the number of non-coding mutations in each sample (referred to as “WGS_coding”, “WGS_non-coding” and “WGS_random_nc” respectively), and tested the performance of our approach on these datasets.

To assess the performance of the MetaWise model on external datasets, we retrieved the somatic mutation data for a prostate cancer cohort^30^ (pcbm_swiss) and a skin melanoma cohort^31^ (skcm_yale) from the cBioPortal database (https://www.cbioportal.org/). The pcbm_swiss cohort consisted of 63 primary and 105 metastatic samples, while the skcm_yale cohort comprised 31 primary and 60 metastatic samples. Additionally, we downloaded paired primary and metastatic whole-exome sequencing (WES) data for 9 treatment-naïve breast cancer patients from the Sequence Read Archive (SRA) database (https://www.ncbi.nlm.nih.gov/sra) to evaluate the model's performance in individual patients^33^. The raw sequencing data were analyzed using the GATK v4.0. pipeline^48^ to accurately identify reliable somatic mutations.

### Mutational signature analysis and data curation

The single-base substitutions were classified into 96 SBS categories considering the 6 substitution types (C > A, C > G, C > T, T > A, T > C and T > G) and their 5’ and 3’ adjacent bases. The doublet bases substitutions were classified into totally 78 DBS categories considering the doublet bases substitution types (AC > NN, AT > NN, CC > NN, CG > NN, CT > NN, GC > NN, TA > NN, TC > NN, TG > NN and TT > NN) and their flanking bases. The small insertion and deletion mutations were classified into 83 ID categories as previous reported considering the insertion or deletion types and the number of repeated lengths^14,49^.

We obtained the SBS, DBS, and ID signatures from COSMIC v3.3 and utilized Sigminer^50^ to analyze the activities of these signatures in the primary and metastatic tumors. A total of 78 SBS, 11 DBS and 18 ID were selected as our proposed input features. To study the potential influence of sequencing artefacts signatures, we generated another feature sets with 89 mutational signatures, including 60 SBS (without sequencing artefacts signatures), 11 DBS and 18 ID signatures. Additionally, to compare with DiaDeL model, we also generated 30 de novo SBS mutational signatures using SigProfiler^14,29^, as detailed in Supplementary Table S6. The exposure matrices of 30 de novo SBS mutational signatures and the 107 COSMIC mutational signatures were applied to the DL models. Further information can be found in Supplementary Table S7, S8.

To study the influence of mutational signature detection procedure and remove potential noise, we employed the percentage of mutations contributed to each signature in each sample as the threshold to filter out mutational signatures. Specifically, we considered a signature to be present in a sample only if a certain percentage (2.5%, 5% or 10%) of mutations contributed to that signature.

### Difference of relative frequency (DRF) analysis of mutational signatures

The difference of relative frequency (DRF) analysis of mutational signatures is adjusted from previous study^18^. We used an equation as below:

$$d\left( {c,s_{i} } \right) = RF\left( {c,s_{i} } \right) - RF\left( {c^{\prime } ,s_{i} } \right),i = 1, \ldots ,30\;{\text{or}}\;1, \ldots ,107$$

where $s_{i}$ represents *i*th signature and $RF(x,s_{i}$) represents the relative frequency of signature $s_{i}$ in primary or metastatic tumors. If $RF(c,s_{i}$) represents the relative frequency of signature $s_{i}$ in primary tumors then $RF(c^{\prime } ,s_{i} )$) represents the relative frequency of $s_{i}$ in the metastatic tumors, and vice versa. We performed this type of analysis in pan-cancer and cancer-type specific manners (in melanoma, breast and prostate cancers) to study the mutational signature profiles between primary and metastatic tumors, respectively.

Neural network architecture and training procedure.

In this research, we aimed to differentiate the mutation profiles of primary and metastatic tumors by developing a ten-layered fully-connected Deep Neural Network (DNN) model using multiple cancer datasets. To further validate our model, we compared it with an existing DL method called DiaDeL^18^. We also experimented with some traditional ML methods such as RF, eXGBoost, SVMs and logistic regression (LR).

Our model was implemented using Keras with Tensorflow^51^ and various parameters such as the learning rate, weight decay, dropout layer, and activation method were fine-tuned using Bayesian optimization^52^, a tool for hyperparameter tuning, to ensure optimal performance. The early stopping strategies was applied to prevent overfitting during the training process. The training was terminated and the best model saved when the validation loss did not decrease for 10 consecutive epochs or started to increase. The best model was defined as the one with the lowest validation loss. We use the recall, accuracy, F1-score, the AUPR and the AUROC to evaluate the performance of each model.

Since our dataset had an imbalanced population of tumors (9740 primary vs 1559 metastasis samples), we randomly divided the primary tumors into five groups and calculated performance metrics based on the predictions made for the validation and test sets through a rigorous internal fivefold cross-validation process. For each fold, the sub-dataset was randomly divided into training, validation, and test sets in an 8:1:1 ratio. The training set was used to train the DL model, the validation set was used to assess the model's performance, and the test set was used to evaluate the model's generalization capability. The same split of data was used for both models to ensure comparability of results. To better understand the model’s prediction, we applied the interpretative ML techniques, SHAP^26^ and LIME^27^, to elucidate the importance and impact of different mutation signatures on the prediction.

## Supplementary Information

Supplementary Figures.

Supplementary Table S1.

Supplementary Table S2.

Supplementary Table S3.

Supplementary Table S4.

Supplementary Table S5.

Supplementary Table S6.

Supplementary Table S7.

Supplementary Table S8.

## Data Availability

All somatic mutations detected by WES of primary and metastatic tumors from TCGA project and other metastatic cohorts are available in cBioPortal database (https://www.cbioportal.org/). The somatic mutations detected by WGS data of PCAWG and POG570 cohort are available in PCAWG website (https://dcc.icgc.org/pcawg) and (https://www.bcgsc.ca/downloads/POG570/), respectively.

## Code availability

The code developed for mutational signatures analysis and CNN model training and testing are available from GitHub: https://github.com/promethiume/MetaWise.
