# Supplementary material for: Deep learning model accurately classifies metastatic tumors from primary tumors based on mutational signatures
Source: Sci Rep. 2023 May 30;13:8752. doi: 10.1038/s41598-023-35842-w (PMC10229594; doi:10.1038/s41598-023-35842-w)
Supplement: Supplementary file 1 — Supplementary Figures. [file 41598_2023_35842_MOESM1_ESM.docx]

**Supplementary figures to:**

Deep Learning model accurately classifies metastatic tumors from primary tumors based on mutational signatures

Weisheng Zheng^1$^(ORCID: 0000-0002-5028-0886), Mengchen Pu^1$^ (ORCID: 0000-0001-6282-2454), Xiaorong Li^1,2^, Zhaolan Du^1,3^, Sutong Jin^1,4^, Xingshuai Li^1^, Jielong Zhou^1^ & Yingsheng Zhang^1*^ (ORCID: 0000-0003-2520-3923)

^1^ StoneWise, AI, Ltd., Haidian District, Beijing, China.

^2^ Minzu University of China, Beijing, China.

^3^ Beijing University of Technology, Beijing, China.

^4^ Harbin Institute of Technology, Weihai, Shandong, China.

^$^ These authors contributed equally: Weisheng Zheng, Mengchen Pu

^*^ Corresponding author

E-mail: [zhangyingsheng@stonewise.cn](mailto:zhangyingsheng@stonewise.cn) (Y. Zhang)

**Supplementary Figure S1. DRF analysis in Melanoma (MEL), Breast Cancer (BC) and Prostate Cancer (PC).**

Difference Relative Frequency (DRF) analysis was performed in melanoma (MEL), breast cancer (BC) and prostate cancer (PC), respectively. DRF scores above 0 indicate a higher degree of enrichment of mutational signatures in primary or metastatic tumors, while DRF scores below 0 suggest the opposite trend.

**Supplementary Figure S2. Disease-specific survival analysis of patients with primary tumors in the test dataset.** Patients with primary tumors in the higher MetaWise-Pan prediction scores (HMPS, misclassified as metastatic tumors) group exhibited significantly lower disease-specific survival compared to those in the lower MetaWise-Pan prediction scores (LMPS, accurately classified as primary tumors) group (p=0.045, log-rank test).


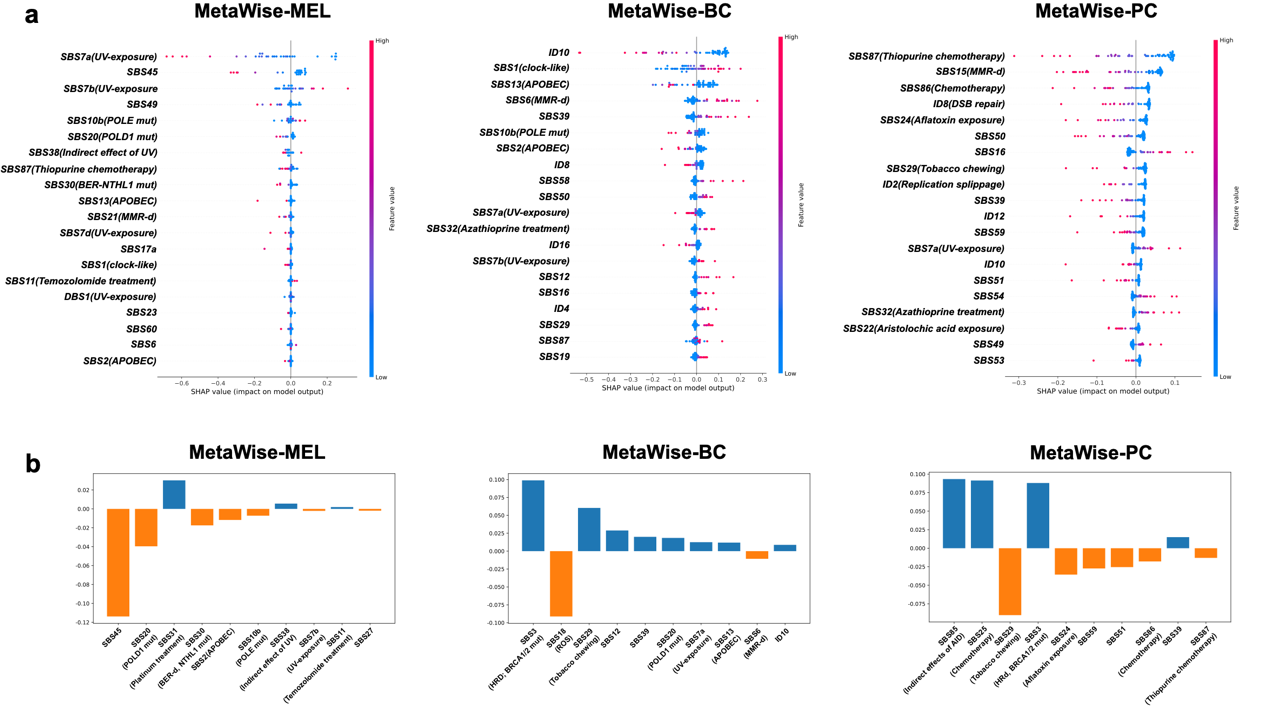


**Supplementary Figure S3. Interpreting the cancer-type-specific models with SHAP and LIME.**

1. Most informative mutational signatures selected by SHAP in MetaWise-MEL (left), MetaWise-BC (mid) and MetaWise-PC (right). The ranking of the signatures is based on the average absolute SHAP value, representing the most important feature for the model. Each dot represents a sample, and the color ranging from blue to red denote the SHAP value from low to high. A more dispersed sample distribution implies a greater impact of the feature, with the x-axis representing the positive or negative influence of the sample's SHAP value. Samples that are both red and have larger positive or negative SHAP values represent a greater positive or negative impact.
2. Most informative mutational signatures selected by LIME in MetaWise-MEL (left), MetaWise-BC (mid) and MetaWise-PC (right). Signatures with positive values impact positively on the model’s prediction, while signatures with negative values impact negatively on the model’s prediction.

**Supplementary Figure S4. SHAP and LIME results in the MetaWise-Mel model with mutational signatures detected at various thresholds.**

1. SHAP results of MetaWise-Mel with mutational signatures detected at 2.5% (left), 5% (middle) and 10% (right) thresholds.
2. LIME results of MetaWise-Mel with mutational signatures detected at 2.5% (left), 5% (middle) and 10% (right) thresholds.

**Supplementary Figure S5. SHAP and LIME results in the MetaWise-BC model with mutational signatures detected at various thresholds.**

1. SHAP results of MetaWise-BC with mutational signatures detected at 2.5% (left), 5% (middle) and 10% (right) thresholds.
2. LIME results of MetaWise-BC with mutational signatures detected at 2.5% (left), 5% (middle) and 10% (right) thresholds.

**Supplementary Figure S6. SHAP and LIME results in the MetaWise-PC model with mutational signatures detected at various thresholds.**

1. SHAP results of MetaWise-PC with mutational signatures detected at 2.5% (left), 5% (middle) and 10% (right) thresholds.
2. LIME results of MetaWise-PC with mutational signatures detected at 2.5% (left), 5% (middle) and 10% (right) thresholds.

**Supplementary Figure S7. Percentage of samples with possible sequencing artefacts signatures in each cohort.**
